# Standard- Versus Reduced-Dose Apixaban and Vitamin K Antagonists in Patients with Atrial Fibrillation and Advanced Chronic Kidney Disease: A Systematic Review and Network Meta-Analysis

**DOI:** 10.3390/jcm15124664

**Published:** 2026-06-16

**Authors:** Bannawich Sapapsap, Wanwarat Aree, Narisa Ruenroengbun, Wichai Santimaleeworagun, Pornwalai Boonmuang

**Affiliations:** 1 Graduate Program in Clinical Pharmacy, Faculty of Pharmacy, Silpakorn University, Nakhon Pathom 73000, Thailand; bannawich1996@gmail.com; 2 Division of Clinical Pharmacy, Faculty of Pharmaceutical Sciences, Burapha University, Chonburi 20131, Thailand; wanwarat.aree@gmail.com; 3 Division of Pharmaceutical Care, Faculty of Pharmacy, Silpakorn University, Nakhon Pathom 73000, Thailand; ruenroengbun_n@su.ac.th (N.R.); santimaleeworag_w@su.ac.th (W.S.); 4 Health Innovation and Research Outcomes (HIRO) Group, Faculty of Pharmacy, Silpakorn University, Nakhon Pathom 73000, Thailand

**Keywords:** apixaban, atrial fibrillation, renal insufficiency, chronic, stroke, hemorrhage

## Abstract

**Background:** The optimal apixaban dose for patients with atrial fibrillation (AF) and advanced chronic kidney disease (CKD; CrCl < 30 mL/min) remains uncertain because randomized trials largely excluded this population, and dose-specific evidence is limited. **Objectives:** This study aimed to compare the efficacy and safety of standard- versus reduced-dose apixaban and to evaluate each regimen against vitamin K antagonists (VKAs). **Methods:** We conducted a systematic review (SR) and network meta-analysis (NMA) that complied with PRISMA 2020. PubMed, Scopus, ScienceDirect, Cochrane Library, and EBSCO Open Dissertations were searched through 10 January 2026. Randomized trials and cohort studies enrolling adults with AF and CrCl < 30 mL/min were included. Primary outcomes were stroke/systemic embolism (SE) and major bleeding; secondary outcomes were any bleeding and all-cause mortality. A frequentist random-effects NMA was conducted using a consistency model. Treatment effects were estimated as hazard ratios (HRs) with 95% confidence intervals (CIs). Pairwise meta-analyses were also performed for direct comparisons. **Results:** Nine studies involving 65,976 patients were included. In the NMA, standard-dose apixaban did not significantly differ from reduced-dose apixaban for stroke/SE, major bleeding, or any bleeding, but was associated with lower all-cause mortality (HR 0.73, 95% CI 0.62–0.87). Compared with VKAs, both standard- and reduced-dose apixaban were associated with lower risks of stroke/SE, major bleeding, any bleeding, and all-cause mortality. **Conclusions:** In AF patients with advanced CKD, both standard- and reduced-dose apixaban were associated with more favorable outcomes than VKAs, although the certainty of evidence was generally low.

## 1. Introduction

Atrial fibrillation (AF) is associated with an approximately fivefold increased risk of stroke [1] and a twofold increase in all-cause mortality [2]. Patients with AF who also have chronic kidney disease (CKD), including both non-end-stage CKD and end-stage kidney disease (ESKD) requiring dialysis, are at particularly high risk of stroke or systemic embolism (SE) and bleeding [3]. This may be related to a prothrombotic state from uremic toxin accumulation, leading to platelet activation and endothelial dysfunction, as well as to vitamin K deficiency associated with dietary restrictions in dialysis patients [4,5,6].

According to the European Society of Cardiology (ESC) guidelines for AF management, oral anticoagulation therapy is recommended for patients with a CHA_2_DS_2_-VA score ≥ 2 points. Direct oral anticoagulants (DOACs) are preferred over vitamin K antagonists (VKAs) such as warfarin because they provide comparable or superior efficacy in preventing stroke and SE, with a lower risk of intracranial hemorrhage except for AF patients with moderate to severe mitral stenosis or mechanical valve replacement for whom warfarin is recommended [7]. However, in AF patients with advanced CKD or those on dialysis, warfarin use is often associated with a suboptimal time in therapeutic range (TTR) [8] and an increased incidence of calciphylaxis [9]. Although DOACs have shown clear advantages over warfarin in patients with normal or moderately impaired renal function, data in patients with advanced renal impairment remain limited. This population includes those with creatinine clearance (CrCl) < 30 mL/min or those receiving renal replacement therapy, who were largely excluded from pivotal randomized controlled trials (RCTs) [10,11]. Nonetheless, the clinical use of DOACs among AF patients with advanced CKD, including those on hemodialysis or peritoneal dialysis, has been steadily increasing [12].

Apixaban, a direct factor Xa inhibitor, is one of the most effective and safest DOACs compared to warfarin for managing patients with non-valvular atrial fibrillation (NVAF), as demonstrated in the large ARISTOTLE trial [13]. Although patients with serum creatinine (Scr) > 2.5 mg/dL or CrCl < 25 mL/min were excluded, apixaban has become the most widely used DOAC in patients with CKD. This is likely due to its relatively low renal elimination of approximately 27% of total clearance and the substantial amount of supporting data across all CKD stages [10,12,14,15]. The clinical practice guideline and the U.S. FDA labeling [16,17] recommend apixaban for patients with advanced renal impairment, including those on dialysis, at a dose of 5 mg twice daily or 2.5 mg twice daily in patients meeting at least two of the following criteria: (1) age ≥ 80 years, (2) body weight ≤ 60 kg, and (3) Scr ≥ 1.5 mg/dL. However, these recommendations are based primarily on small pharmacokinetic (PK) and pharmacodynamic (PD) studies [18,19]. Conversely, the 2021 European Heart Rhythm Association (EHRA) Practical Guide recommends apixaban 2.5 mg twice daily for patients with CrCl < 30 mL/min and advises against the use of any DOAC in those with CrCl < 15 mL/min, owing to persistent uncertainty regarding efficacy and safety in this population [11].

Previous systematic reviews (SR) and meta-analyses (MA) [20,21], as well as network meta-analyses (NMA) [22,23,24], have evaluated anticoagulation strategies in patients with AF and advanced CKD; however, most focused on comparisons between DOACs, VKAs, and no oral anticoagulation, or were restricted to dialysis populations, without specifically addressing dose-stratified apixaban outcomes. Contemporary evidence also remains inconsistent. Some observational studies have suggested that standard-dose apixaban may provide greater protection against stroke/SE than reduced-dose apixaban [25], whereas others reported no significant differences between dosing strategies, particularly among dialysis patients [26]. Similarly, comparisons with VKAs have yielded conflicting findings regarding whether potential benefits are dose-dependent [25,27]. These discrepancies underscore the need for a focused dose-specific synthesis in this high-risk population.

Therefore, we performed an SR-NMA to compare and rank the efficacy and safety of standard- and reduced-dose apixaban with those of VKAs in patients with AF and advanced CKD.

## 2. Methods

### 2.1. Search Strategy

This SR-NMA was conducted in accordance with the Preferred Reporting Items for Systematic Reviews and Meta-Analyses (PRISMA) 2020 statement [28] and the PRISMA extension statement for reporting of systematic reviews incorporating network meta-analyses (PRISMA-NMA) (Supplementary File S1). The protocol was registered in PROSPERO (CRD420261340028).

A comprehensive literature search was performed in PubMed, Scopus, ScienceDirect, and the Cochrane Library. In addition, grey literature was identified through EBSCO Open Dissertations. The search included all records published up to 10 January 2026. The search strategy combined Medical Subject Headings (MeSH) terms and free-text keywords related to the population (e.g., “atrial fibrillation,” “renal insufficiency”), interventions (e.g., “apixaban,” “vitamin K antagonists”), and outcomes (e.g., “stroke,” “hemorrhage”). Boolean operators (AND, OR) were used to combine search concepts. The complete search strategy for each database is provided in Supplementary Table S1. No language or time restrictions were imposed.

### 2.2. Study Eligibility Criteria

We included RCTs and cohort studies that met the following criteria: (1) adult patients with AF and advanced CKD, defined as CrCl < 30 mL/min, including both dialysis and non-dialysis populations; (2) treatment with apixaban or VKAs for stroke prevention; and (3) reporting at least one clinical outcome of interest, including stroke or SE, or major bleeding. Studies were excluded if they enrolled mixed populations of AF and VTE without separate AF-specific analyses, or if they compared apixaban with VKAs without providing dose-specific outcome data for apixaban. Two investigators (B.S. and W.A.) independently screened titles, abstracts, and full-text articles for eligibility. Disagreements were resolved through discussion and consultation with senior authors (N.R., W.S., and P.B.).

### 2.3. Data Extraction

Two investigators (B.S. and W.A.) independently extracted data using a standardized form in Microsoft Excel^®^. The following study characteristics were collected: study design, study period, sample size, follow-up duration, study location, and baseline patient characteristics, including sex, age, renal function, dialysis status, CHA_2_DS_2_-VASc score, HAS-BLED score, history of stroke or systemic embolism, and prior bleeding. Treatment-related variables included apixaban regimen, dose-reduction criteria, use of a comparator VKA, international normalized ratio (INR) target range, TTR, comorbidities, and concomitant medications. The primary outcomes were stroke or systemic embolism and major bleeding. Secondary outcomes included any bleeding and all-cause mortality. Outcome definitions were recorded as reported in each study. Disagreements were resolved, when necessary, through consultation with senior authors (N.R., W.S., and P.B.). Authors were contacted for missing data when necessary.

### 2.4. Risk of Bias Assessment

Risk of bias was assessed independently by two investigators (B.S. and W.A.) using the Cochrane Risk of Bias Tool version 2.0 (RoB 2.0) for RCTs [29] and the Risk Of Bias In Non-randomized Studies of Interventions (ROBINS-I) for observational studies [30]. The overall risk was determined by the highest level of bias across domains. Disagreements were resolved through discussion and, when necessary, consultation with senior authors (N.R., W.S., and P.B.). Results were visualized using the ROBVIS tool [31].

### 2.5. Statistical Analysis

A frequentist random-effects network meta-analysis (NMA) was conducted in accordance with the PRISMA extension for network meta-analyses [32] to simultaneously compare the efficacy and safety of three treatment strategies: standard-dose apixaban (5 mg twice daily), reduced-dose apixaban (2.5 mg twice daily), and VKAs. Treatment effects were estimated as hazard ratios (HRs) with 95% confidence intervals (CIs). For studies reporting adjusted HRs, log-transformed HRs, and their standard errors were entered directly. For studies reporting adjusted relative risks (RRs) or odds ratios (ORs), these were approximated as HRs given the low event rates observed. Between-study heterogeneity was accounted for using a random-effects model. Consistency between direct and indirect evidence was assessed using the design-by-treatment interaction model. The network’s transitivity assumption was assessed by examining the distribution of key clinical and methodological characteristics across treatment comparisons. These included age, sex, body weight, race, CKD stage, CHA_2_DS_2_-VASc score, HAS-BLED score, history of prior bleeding or stroke, comorbid conditions, and concomitant antiplatelet therapy. Treatments were ranked for each outcome using the surface under the cumulative ranking curve (SUCRA). To facilitate interpretation of the balance between efficacy and safety, SUCRA estimates were subsequently visualized using quadrant plots comparing thromboembolic outcomes with major bleeding and mortality with any bleeding. The certainty of evidence for each network estimate was appraised using the Confidence in Network Meta-Analysis (CINeMA) framework [33]. Potential publication bias within the network was explored using adjusted funnel plots. All network analyses were performed using STATA^®^ version 18.0 (StataCorp LLC, College Station, TX, USA).

In addition, conventional pairwise meta-analyses were conducted to evaluate direct comparisons between treatments. Adjusted effect estimates (HRs, RRs, or ORs) with 95% CIs were preferentially extracted; when only crude event counts were available, unadjusted estimates were calculated using the inverse-variance method. Random-effects models with inverse-variance weighting were applied. Fixed-effect models were used when heterogeneity was minimal (I^2^ < 25%). Statistical heterogeneity was quantified using Cochran’s Q test and the I^2^ statistic, with Q-test *p*-values < 0.10 considered indicative of heterogeneity. I^2^ values greater than 75% were interpreted as high heterogeneity, whereas values below 25% indicated low heterogeneity [34]. Prespecified subgroup analyses were conducted according to study design (RCT vs. observational studies) and dialysis status (dialysis vs. non-dialysis). Sensitivity analyses excluded studies without adjusted effect estimates. Pairwise analyses were performed using Review Manager (RevMan) version 5.4 (The Nordic Cochrane Centre, Copenhagen, Denmark), and a two-sided *p*-value < 0.05 was considered statistically significant.

## 3. Results

### 3.1. Search Results

**Figure 1:**
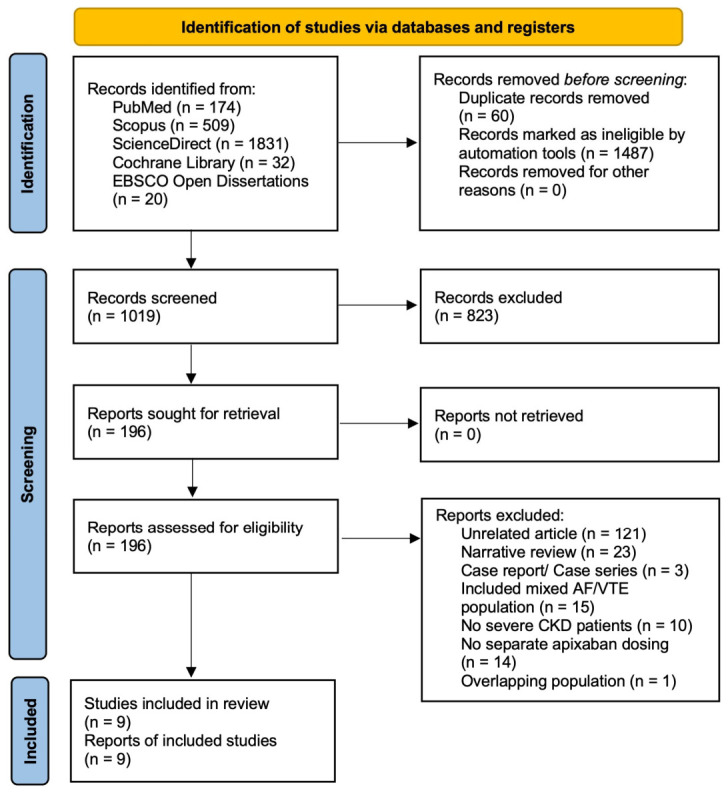
PRISMA flow diagram of study selection.

The database search across five electronic sources identified 2566 records. After removing duplicates using EndNote and records marked as ineligible by automated tools, 1019 unique records remained. Following title and abstract screening, 196 articles underwent full-text review. After applying the eligibility criteria, nine studies met the inclusion criteria and were included in the SR-NMA. The study selection process is illustrated in Figure 1.

### 3.2. Study Characteristics

Among the nine included studies, two were RCTs [35,36], and seven were non-RCT studies [25,26,27,37,38,39,40]. The total sample comprised 65,976 patients, including 20,796 treated with apixaban and 45,180 receiving VKAs. Approximately 55% of participants were male. Overall, most studies were conducted in Western countries, predominantly in the United States. The mean age across studies ranged from 66 to 80 years (overall mean approximately 71 years). The average CHA_2_DS_2_-VASc score was 4.89, and the mean HAS-BLED score was 2.90. A history of prior stroke or systemic embolism was reported in 28.41% of patients, while 15.47% had a history of prior bleeding. Median follow-up duration ranged from 8 months to 3.0 years across studies. Detailed baseline characteristics are presented in Table 1. Information regarding apixaban regimens, dose-reduction criteria, VKA comparators, INR target ranges, and TTR is summarized in Supplementary Table S2. Comorbidities, concomitant medications, and outcome definitions are provided in Supplementary Tables S3 and S4, respectively.

### 3.3. Risk of Bias Assessment

Among the two RCTs, one was judged to have a low risk of bias [36], while the other was rated as having some concerns [35] according to the RoB 2.0 tool. The judgment was primarily driven by concerns regarding selective reporting (Domain 5), as the subgroup analysis by CrCl was conducted post hoc and not prespecified. The detailed RoB 2.0 assessments are presented in Supplementary Figure S1. According to the ROBINS-I tool, all included observational studies were judged to have an overall moderate risk of bias [25,26,27,37,38,39,40], primarily due to the potential for residual confounding inherent to non-randomized study designs. The detailed ROBINS-I assessments are presented in Supplementary Figure S2.

### 3.4. Stroke or Systemic Embolism Outcomes

**Figure 2:**
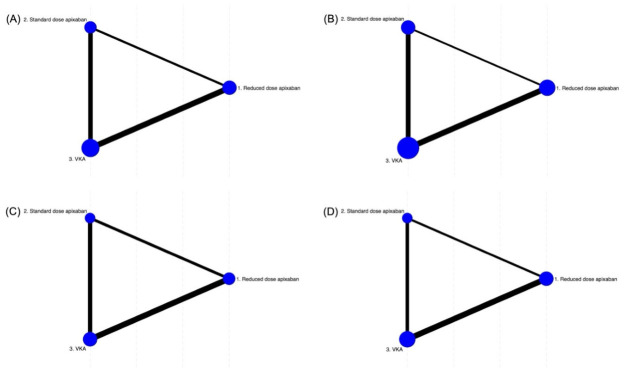
Network plots of treatment comparisons for (**A**) stroke or systemic embolism, (**B**) major bleeding, (**C**) any bleeding, and (**D**) all-cause mortality.

In the NMA, standard-dose apixaban did not differ significantly from reduced-dose apixaban in preventing stroke or systemic embolism (HR 1.05, 95% CI 0.66–1.68). However, both apixaban regimens were associated with significantly lower risks of stroke/SE compared with VKAs (standard-dose: HR 0.59, 95% CI 0.39–0.89; reduced-dose: HR 0.56, 95% CI 0.37–0.85). The network structure is presented in Figure 2, and detailed estimates are shown in Table 2 and Supplementary Table S5. Treatment ranking based on SUCRA indicated that reduced-dose apixaban had the highest probability of being the most effective strategy, followed by standard-dose apixaban, whereas VKAs ranked last (Supplementary Table S6; Supplementary Figure S3). The certainty of evidence for most comparisons ranged from low to very low, primarily due to concerns related to within-study bias, imprecision, and heterogeneity (Supplementary Table S7).

In the pairwise meta-analysis of direct comparisons, standard-dose apixaban showed a numerically lower risk of stroke/SE than reduced-dose, without statistical significance (HR 0.78, 95% CI 0.55–1.10; I^2^ = 45%). Compared with VKAs, only standard-dose apixaban was associated with a significantly lower risk of stroke/SE (Supplementary Figure S4). Sensitivity and subgroup analyses yielded results consistent with the primary findings (Supplementary Tables S8 and S9).

### 3.5. Major Bleeding Outcomes

**Figure 3:**
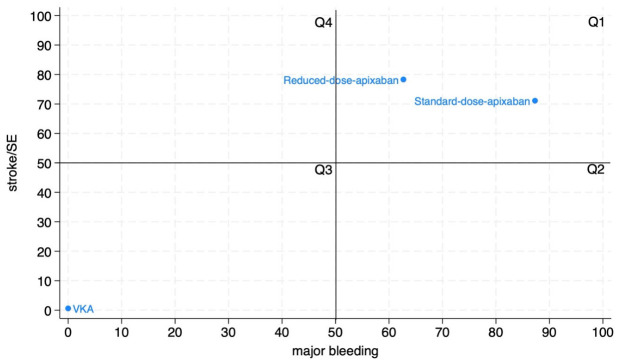
Quadrant plot comparing treatments based on the surface under the cumulative ranking curve (SUCRA) for stroke or systemic embolism (*y*-axis) and major bleeding (*x*-axis). Quadrant interpretation: Q1 indicates favorable efficacy and safety; Q2 indicates less favorable efficacy but better safety; Q3 indicates less favorable efficacy and safety; and Q4 indicates better efficacy but less favorable safety. SE: systemic embolism.

In the NMA, no statistically significant difference in major bleeding was observed between standard- and reduced-dose apixaban (HR 0.95, 95% CI 0.81–1.12). In contrast, both dosing regimens were associated with significantly lower risks of major bleeding compared with VKAs (standard-dose: HR 0.54, 95% CI 0.47–0.61; reduced-dose: HR 0.56, 95% CI 0.50–0.64). The network structure is shown in Figure 2, with detailed estimates provided in Table 2 and Supplementary Table S5. Consistent with the effect estimates, SUCRA ranking placed standard-dose apixaban first, followed by reduced-dose apixaban, whereas VKAs ranked lowest (Supplementary Table S6; Supplementary Figure S3). When SUCRA estimates were visualized using quadrant plots comparing stroke/SE prevention with major bleeding, both apixaban regimens were located in the favorable quadrant, indicating a balanced efficacy-safety profile. Reduced-dose apixaban showed slightly stronger efficacy for thromboembolic prevention, whereas standard-dose apixaban demonstrated a marginal advantage in bleeding reduction. In contrast, VKAs were located in the unfavorable quadrant, indicating poorer performance on both outcomes (Figure 3). The certainty of the evidence for these comparisons was rated as very low, primarily due to concerns about within-study bias, imprecision, and heterogeneity (Supplementary Table S7).

In pairwise analyses, no significant difference in major bleeding was observed between standard- and reduced-dose apixaban (HR 1.08, 95% CI 0.90–1.29; I2 = 0%), while both regimens showed lower bleeding risk than VKAs (Supplementary Figure S4). Sensitivity and subgroup analyses yielded results consistent with the primary findings (Supplementary Tables S8 and S9).

### 3.6. Any Bleeding Outcomes

In the NMA, standard- and reduced-dose apixaban did not differ significantly in the risk of any bleeding (HR 1.07, 95% CI 0.90–1.28). However, both regimens were associated with significantly lower risks of any bleeding compared with VKAs (standard-dose: HR 0.63, 95% CI 0.54–0.73; reduced-dose: HR 0.59, 95% CI 0.51–0.67). The network structure is shown in Figure 2, and detailed estimates are provided in Supplementary Table S5. SUCRA rankings were consistent with these findings, indicating that reduced-dose apixaban had the highest probability of being the safest strategy, followed by standard-dose apixaban, while VKAs ranked lowest (Supplementary Table S6; Supplementary Figure S3). The certainty of evidence was generally rated as moderate, although one comparison was graded as very low due to concerns related to within-study bias and imprecision (Supplementary Table S7).

In pairwise analyses, no significant difference was observed between dosing strategies (HR 1.10, 95% CI 0.47–2.58, most points located in the upper funnel region; I^2^ = 76%). Compared with VKAs, only standard-dose apixaban demonstrated a statistically significant reduction in any bleeding (Supplementary Figure S4). Sensitivity and subgroup analyses yielded findings consistent with the primary results (Supplementary Tables S8 and S9).

### 3.7. All-Cause Mortality Outcomes

**Figure 4:**
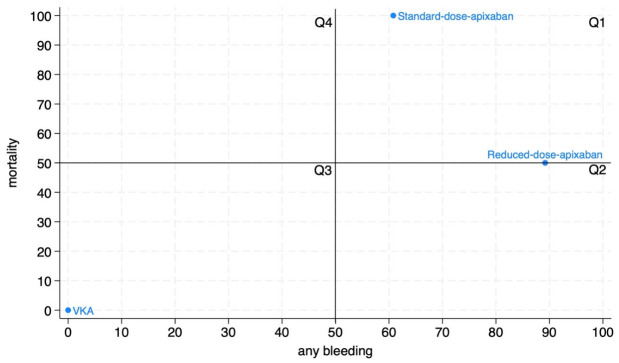
Quadrant plot comparing treatments based on the surface under the cumulative ranking curve (SUCRA) for all-cause mortality (*y*-axis) and any bleeding (*x*-axis). Quadrant interpretation: Q1 indicates favorable efficacy and safety; Q2 indicates less favorable efficacy but better safety; Q3 indicates less favorable efficacy and safety; and Q4 indicates better efficacy but less favorable safety.

In the NMA, standard-dose apixaban was associated with a significantly lower risk of all-cause mortality compared with reduced-dose apixaban (HR 0.73, 95% CI 0.62–0.87). Both apixaban regimens were also associated with significantly lower mortality than VKAs (standard-dose: HR 0.55, 95% CI 0.47–0.64; reduced-dose: HR 0.75, 95% CI 0.65–0.86). The network structure is shown in Figure 2, with detailed estimates provided in Supplementary Table S5. SUCRA rankings were consistent with these findings, indicating that standard-dose apixaban had the highest probability of being the most favorable strategy, followed by reduced-dose apixaban, while VKAs ranked lowest (Supplementary Table S6; Supplementary Figure S3). When SUCRA estimates were visualized using quadrant plots comparing mortality with any bleeding, both apixaban regimens were positioned in the favorable quadrant, indicating balanced efficacy and safety. Standard-dose apixaban showed the most favorable ranking for all-cause mortality in the network estimates, whereas reduced-dose apixaban showed slightly better performance for bleeding outcomes. VKAs were located in the unfavorable quadrant, reflecting poorer overall performance (Figure 4). The certainty of the evidence ranged from moderate to low, mainly due to concerns about within-study bias and heterogeneity (Supplementary Table S7).

In pairwise analyses, no significant difference in mortality was observed between standard- and reduced-dose apixaban. Only standard-dose apixaban showed a trend toward lower mortality compared with VKAs (HR 0.87, 95% CI 0.76–1.00) (Supplementary Figure S4). Sensitivity and subgroup analyses yielded results consistent with the primary findings (Supplementary Tables S8 and S9).

### 3.8. Inconsistency, Transitivity, and Publication Bias

Across all outcomes, inconsistency tests yielded *p*-values > 0.05, indicating no evidence of disagreement between direct and indirect comparisons (Supplementary Table S10). Baseline characteristics across treatment comparisons were generally comparable, supporting the plausibility of the transitivity assumption. A descriptive comparison of key baseline covariates is presented in Supplementary Table S11. Potential publication bias in the network meta-analysis was explored using adjusted funnel plots. The distribution of study estimates was symmetrical, with no clear evidence of small-study effects (Supplementary Figure S5). For pairwise meta-analyses, publication bias was evaluated using funnel plots for the major bleeding outcome, which included the largest number of contributing studies. The distribution of studies appeared broadly symmetrical, suggesting a low likelihood of publication bias (Supplementary Figure S6). Funnel plots were not generated for other outcomes due to the limited number of available studies.

## 4. Discussion

In this SR-NMA, we compared the efficacy and safety of standard- and reduced-dose apixaban in patients with AF and advanced CKD (CrCl < 30 mL/min) and evaluated each regimen against VKAs. To our knowledge, this study is among the first to specifically assess dose-stratified apixaban outcomes in this high-risk population. Overall, standard- and reduced-dose apixaban showed largely similar clinical outcomes. A significant difference was observed only for all-cause mortality in the network analysis, where standard-dose apixaban was associated with lower mortality than reduced-dose apixaban. Both apixaban regimens, however, were associated with more favorable efficacy and safety profiles than VKAs across all outcomes. In contrast, direct pairwise analyses did not identify significant differences between dosing strategies, and subgroup analyses by dialysis status showed no meaningful differences in thromboembolic or major bleeding outcomes. This may partly reflect the pharmacokinetic profile of apixaban, which is minimally removed by hemodialysis (approximately 4–7%) [18,19]. The stronger statistical signals observed in the network analysis likely reflect the integration of both direct and indirect evidence, which increases the overall precision of treatment comparisons. The CINeMA assessment indicated certainty of evidence ranging from moderate to very low, mainly due to within-study bias from observational designs, as well as imprecision and heterogeneity. Although no important inconsistency was identified, and the assessment of transitivity together with the overall comparability of baseline characteristics across studies supported the validity of the network findings, residual heterogeneity may still exist, particularly regarding dialysis prevalence, CKD severity, and variability in dose-reduction practices, which may introduce some limitations to the transitivity assumption.

The lack of clear differences between dosing strategies may reflect underlying clinical and methodological heterogeneity. Study populations varied across CKD stages, baseline risks, outcome definitions, and follow-up durations. From a mechanistic perspective, advanced CKD is associated with alterations in drug exposure [18,19] and hemostatic balance [4,5,6], which may further complicate the relationship between apixaban dose and both thromboembolic and bleeding outcomes. Most data were derived from observational studies, in which dose selection is influenced by clinical judgment, potentially introducing confounding by indication. In some studies, reduced-dose apixaban was used outside recommended criteria [26], further contributing to residual confounding. Additionally, the limited number of direct comparisons may have reduced statistical power to detect meaningful differences.

When the SUCRA rankings were visualized using quadrant plots, both apixaban dosing regimens were located in the first quadrant, suggesting favorable efficacy and safety profiles. However, standard-dose apixaban ranked slightly higher for major bleeding reduction and all-cause mortality compared with the reduced-dose regimen. This discrepancy may reflect heterogeneity in bleeding definitions and real-world prescribing patterns, where patients receiving reduced-dose apixaban are often older, frailer, or at higher baseline bleeding risk, thereby potentially attenuating its apparent benefit [25,26,27,39]. In contrast, VKAs consistently ranked lowest, indicating a less favorable overall clinical profile. These findings align with prior evidence demonstrating improved efficacy and safety of apixaban compared with warfarin across a range of renal function [13,20,21]. However, these results should be interpreted cautiously, given the predominance of observational evidence and variability in VKA management, particularly differences in TTR. In addition, all-cause mortality in advanced CKD populations is influenced by multiple competing non-cardiovascular risks, including infection, frailty, and dialysis-related complications, which may further limit causal interpretation of anticoagulation-related mortality differences.

Our finding that standard-dose apixaban was associated with lower mortality than reduced-dose apixaban is consistent with a previous network meta-analysis [24], although that estimate was largely driven by a single observational study [25]. In contrast, two other studies reported no significant differences in thromboembolic, bleeding, or mortality outcomes when comparing standard- and reduced-dose apixaban [22,23]. When compared with VKAs, our results are broadly consistent with prior meta-analyses showing lower risks of stroke and major bleeding with apixaban, without a clear mortality benefit [20,21]. Another study similarly reported a reduction in major bleeding but did not demonstrate a significant benefit in thromboembolic outcomes compared with warfarin [41]. Previous network meta-analyses also reported lower major bleeding with both dosing regimens compared with VKAs [23,24], while some suggested a mortality benefit with standard-dose apixaban only [22,24], a pattern consistent with our findings. Unlike prior analyses, which often included mixed AF and VTE populations [20,21,22,23,41] or were limited to dialysis cohorts [20,21,22,23,24], our study focused exclusively on AF with advanced CKD (CrCl < 30 mL/min), including both dialysis and non-dialysis populations. To our knowledge, this is the first meta-analysis to directly compare standard- and reduced-dose apixaban while concurrently evaluating both regimens against VKAs in this population.

Several limitations should be acknowledged. First, most included studies were conducted in Western populations, predominantly in the United States, which may limit generalizability. Second, the majority of included studies were observational cohorts; therefore, residual confounding cannot be fully excluded despite the use of adjusted estimates. In the pairwise meta-analyses, adjusted effect measures were preferentially used; however, two studies reported only crude event data [36,37]. Sensitivity analyses excluding these studies yielded results consistent with the primary findings. Third, variability in dose classification and incomplete reporting of key variables, particularly TTR in VKA cohorts, dose-reduction criteria, and concomitant antiplatelet therapy, may have substantially influenced comparative outcome estimates. Fourth, moderate-to-high heterogeneity was observed in several pairwise meta-analyses, particularly for the any bleeding outcomes, likely reflecting differences in study design, patient characteristics, follow-up duration, and outcome definitions. Fifth, the absence of patient-level data precluded risk-stratified analyses, such as bleeding risk or dialysis modality (hemodialysis vs peritoneal dialysis), and pooled estimates should therefore be interpreted cautiously. Finally, given the predominance of observational evidence, the findings should be interpreted as associations rather than causal effects.

Although standard-dose apixaban appeared to provide a mortality benefit compared with the reduced dose, differences in other outcomes were less consistent. Importantly, even in patients with advanced CKD, both apixaban regimens (5 mg and 2.5 mg) were associated with more favorable outcomes than VKAs across evaluated endpoints. Therefore, in current clinical practice, apixaban dose selection should remain individualized based on patient-specific factors, such as renal function, age, and body weight [13,17], rather than assuming that one dosing strategy is universally preferable.

## 5. Conclusions

In patients with atrial fibrillation and advanced CKD (CrCl < 30 mL/min), both standard- and reduced-dose apixaban were associated with more favorable clinical outcomes than VKAs. While most outcomes were comparable between dosing strategies, standard-dose apixaban was associated with lower all-cause mortality in the network estimates; however, this finding should be interpreted cautiously and considered hypothesis-generating, given the potential for residual confounding and dose-selection bias inherent in observational studies. Individualized dose selection remains important, and further well-designed randomized or large prospective studies are required to define the optimal dosing strategy.

## Figures and Tables

**Table 1:** Characteristics of the included studies.

Author, Year	Study Design	Study Period	Analyzed Sample Size (N)	Follow-Up Duration	Male, N (%)	Age, Years ^a^	BW (kg) or BMI ^a^	Renal Function	CHA_2_DS_2_-VASc Score ^a^	HAS-BLED Score ^a^	Prior Stroke/SE, N (%)	Prior Bleeding, N (%)	Location
Siontis 2018 [25]	Retrospective cohort study	2010– 2015	25,523 (Apix 2351, VKA 23,172)	Average time on treatment: Apix = 105 days VKA = 157 days	13,852 (54.3)	68.22 ± 11.89	N/A	Stage 5 Dialysis	5.24 ± 1.79	N/A	8461 (33.2)	Major bleeding: 2536 (9.9) GI bleeding: 2966 (11.6)	United States
Stanifer 2020 [35]	Double-blind randomized controlled trial ^b^	2006–2010	269 (Apix 136, VKA 133)	1.8 years	106 (39.4)	80.65 ± 6.71	BW 57.00 ± 11.93	Stage 4 Non-dialysis	4.80 ± 1.42	2.30 ± 1.05	73 (27.1)	N/A	Multicenter study across 39 countries (North America, Latin America, Europe, Asia-Pacific)
Elis 2021 [37]	Sub-analysis of a multicenter prospective cohort study	2014–2017	152 (Apix 76, VKA 76)	1 year	81 (53.3)	80.58 ± 9.81	N/A	Stage 4 Non-dialysis	5.13 ± 1.40	2.49 ± 1.06	18 (11.8)	12 (7.9)	Israel
Fu CM 2021 [38]	Retrospective cohort study	2004–2018	3250 (Apix 1625, VKA 1625)	N/A ^c^	1864 (57.4)	74–75 (NR)	N/A	Stage 4–5 Non-dialysis	3.82 ± 1.69	2.92 ± 1.35	1030 (31.7)	963 (29.6)	Taiwan
Wetmore 2022 [26]	Retrospective cohort study	2013–2018	17,156 (Apix 4639, VKA 12,517)	567 days	10,585 (61.7) ^d^	66.20 ± 9.40	N/A	Stage 5 Dialysis	4.50 ± 1.70	3.00 ± 0.80	21.2% ^d^	N/A	United States
Reinecke 2023 [36]	Prospective randomized open blinded endpoint	2017–2022	97 (Apix 48, VKA 49)	Median (Q1 and Q3), 462 (253–702) days	68 (70.1)	74.70 ± 7.90	BMI 28.60 ± 6.10	Stage 5 Dialysis	4.52 ± 1.55	4.20 ± 1.02	N/A	N/A	Germany
Xu 2023 [39]	Retrospective cohort study	2013–2021	4313 (Apix 5 mg 1705, Apix 2.5 mg 2608)	Median (IQR), 8 (4–15) months	43.0% ^d^	77.00 ± 9.00	BW 87.00 ± 24.50	Stage 4–5 Non-dialysis	3.70 ± 1.60	2.50 ± 1.00	34.4% ^d^	3.8% ^d^	United States
Fu 2024 [40]	Retrospective cohort study	2013– 2022	12,488 (Apix 6244, VKA 6244)	Mean (SD) 276.6 (332.4) days	6324 (50.6)	78.75 ± 7.60	N/A	Stage 4–5 Non-dialysis	5.38 ± 1.50	2.91 ± 0.67	3546 (28.40)	3400 (27.23)	United States
Wu 2025 [27]	Retrospective cohort study	2017–2023	2728 (Apix 1364, VKA 1364)	The median duration of follow-up 2.7 to 3.0 years	1626 (59.6)	70.40 ± 10.50	BMI 30.65 ± 7.10	Stage 5 with or without dialysis	N/A	N/A	464 (17.0)	N/A	TriNetX Global Collaborative Network, 104 health care organizations in 15 countries

^a^ Data reported as mean ± SD unless otherwise specified, ^b^ Subgroup analysis of the ARISTOTLE trial in patients with creatinine clearance 25–30 mL/min, ^c^ Follow-up duration not reported, censoring at first event, treatment discontinuation/switch, loss to follow-up ≥ 365 days, or database cut-off (31 December 2018). ^d^ Estimated by the authors based on available data. Apix: apixaban, BMI: body mass index, BW: body weight, IQR: interquartile range, N/A: not applicable, SD: standard deviation, SE: systemic embolism, VKA: vitamin K antagonist.

**Table 2:** League table of the network meta-analysis comparing standard-dose apixaban, reduced-dose apixaban, and vitamin K antagonists for stroke/systemic embolism and major bleeding.

		Stroke/SE, HR (95% CI)
**Standard-dose apixaban**	1.05 (0.66–1.68)	0.59 (0.39–0.89) *
0.95 (0.81–1.12)	**Reduced-dose apixaban**	0.56 (0.37–0.85) *
0.54 (0.47–0.61) *	0.56 (0.50–0.64) *	**VKA**
Major bleeding, HR (95% CI)		

* Statistically significant difference CI: confidence interval, HR: hazard ratio, SE: systemic embolism, VKA: vitamin K antagonist.

## Data Availability

The raw data supporting the conclusions of this article will be made available by the authors upon reasonable request.

## Supplementary Materials

The following supporting information can be downloaded at https://www.mdpi.com/article/10.3390/jcm15124664/s1. File S1: PRISMA NMA Checklist; Figure S1: Risk of bias assessment of randomized controlled trials using the Cochrane Risk of Bias 2 (RoB 2) tool; Figure S2: Risk of bias assessment of non-randomized studies using the ROBINS-I tool; Figure S3: Surface under the cumulative ranking curve (SUCRA) plots for treatment ranking; Figure S4: Forest plots from pairwise meta-analyses comparing treatment regimens; Figure S5: Comparison-adjusted funnel plots from the network meta-analysis; Figure S6: Funnel plot of pairwise meta-analyses; Table S1: Search strategy; Table S2: Intervention and comparator regimens; Table S3: Comorbidities and concomitant medications of included studies; Table S4: Definitions of outcomes; Table S5: Network meta-analysis estimates for stroke or systemic embolism, major bleeding, any bleeding, and all-cause mortality; Table S6: Surface under the cumulative ranking curve (SUCRA) values; Table S7: Confidence in Network Meta-Analysis (CINeMA) assessment; Table S8: Sensitivity analysis excluding studies reporting crude event data (pairwise meta-analysis); Table S9: Subgroup analysis results from the pairwise meta-analysis; Table S10: Results of inconsistency testing in the network meta-analysis; Table S11: Baseline characteristics of included studies relevant to the assessment of transitivity.

## Abbreviations

The following abbreviations are used in this manuscript:

AF	Atrial fibrillation
CINeMA	Confidence in Network Meta-Analysis
CKD	Chronic kidney disease
CrCl	Creatinine clearance
DOAC	Direct oral anticoagulant
INR	International normalized ratio
PRISMA	Preferred Reporting Items for Systematic Reviews and Meta-Analyses
RCT	Randomized controlled trial
SE	Systemic embolism
SR-NMA	Systematic review and network meta-analysis
SUCRA	Surface under the cumulative ranking curve
TTR	Time in therapeutic range
VKA	Vitamin K antagonist
VTE	Venous thromboembolism
