# Supplementary material for: Standard- Versus Reduced-Dose Apixaban and Vitamin K Antagonists in Patients with Atrial Fibrillation and Advanced Chronic Kidney Disease: A Systematic Review and Network Meta-Analysis
Source: J Clin Med. 2026 Jun 16;15(12):4664. doi: 10.3390/jcm15124664 (PMC13301731; doi:10.3390/jcm15124664)

Supplementary Table

Table S1. Search strategy

Table S2. Intervention and comparator regimens

Table S3. Comorbidities and concomitant medications of included studies

Table S4. Definitions of outcomes

Table S5. Network meta-analysis estimates for stroke or systemic embolism, major bleeding, any bleeding, and all-cause mortality

Table S6. Surface under the cumulative ranking curve (SUCRA) values

- Table S6.1 Stroke or systemic embolism
- Table S6.2 Major bleeding
- Table S6.3 Any bleeding
- Table S6.4 All-cause mortality

Table S7. Confidence in Network Meta-Analysis (CINeMA) assessment

- Table S7.1 Stroke or systemic embolism
- Table S7.2 Major bleeding
- Table S7.3 Any bleeding
- Table S7.4 All-cause mortality

Table S8. Sensitivity analysis excluding studies reporting crude event data (pairwise meta-analysis)

Table S9. Subgroup analysis results from the pairwise meta-analysis

Table S10. Results of inconsistency testing in the network meta-analysis

Table S11. Baseline characteristics of included studies relevant to the assessment of transitivity

Supplementary Figures

Figure S1. Risk of bias assessment of randomized controlled trials using the Cochrane Risk of Bias 2 (RoB 2) tool

Figure S2. Risk of bias assessment of non-randomized studies using the ROBINS-I tool

Figure S3. Surface under the cumulative ranking curve (SUCRA) plots for treatment ranking

- Figure S3.1 Stroke or systemic embolism
- Figure S3.2 Major bleeding
- Figure S3.3 Any bleeding
- Figure S3.4 All-cause mortality

Figure S4. Forest plots from pairwise meta-analyses comparing treatment regimens

- Figure S4.1 Stroke or systemic embolism comparing
  (A) standard-dose apixaban versus reduced-dose apixaban,
  (B) standard-dose apixaban versus VKAs, and
  (C) reduced-dose apixaban versus VKAs
- Figure S4.2 Major bleeding comparing
  (A) standard-dose apixaban versus reduced-dose apixaban,
  (B) standard-dose apixaban versus VKAs, and
  (C) reduced-dose apixaban versus VKAs
- Figure S4.3 Any bleeding comparing
  (A) standard-dose apixaban versus reduced-dose apixaban,
  (B) standard-dose apixaban versus VKAs, and
  (C) reduced-dose apixaban versus VKAs
- Figure S4.4 All-cause mortality comparing
  (A) standard-dose apixaban versus reduced-dose apixaban,
  (B) standard-dose apixaban versus VKAs, and
  (C) reduced-dose apixaban versus VKAs

Figure S5. Comparison-adjusted funnel plots from the network meta-analysis

- Figure S5.1 Stroke or systemic embolism
- Figure S5.2 Major bleeding
- Figure S5.3 Any bleeding
- Figure S5.4 All-cause mortality

Figure S6. Funnel plot of pairwise meta-analyses

Table S1. Search strategy

Date: inception to 10 January 2026

Database	Search term
PubMed (174)	("Apixaban"[Supplementary Concept] OR apixaban OR BMS562247 OR BMS-562247 OR Eliquis) AND ("antivitamins K" [Supplementary Concept] OR “antivitamins K” OR “vitamin K antagonists” OR "Warfarin"[Mesh] OR warfarin OR coumadin OR "phenprocoumon"[Mesh] OR phenprocoumon OR phenprocoumarol) AND ("stroke"[Mesh] OR stroke OR “cerebrovascular accident” OR "hemorrhage"[Mesh] OR hemorrhage OR bleeding OR epistaxis OR hematemesis OR melena) AND ("Atrial Fibrillation"[Mesh] OR "Atrial Fibrillation") AND ("Renal Insufficiency, Chronic"[Mesh] OR “chronic renal insufficiency” OR “chronic kidney insufficiency” OR “chronic kidney disease” OR “chronic renal disease” OR “end-stage kidney disease” OR “end-stage renal disease” OR “end-stage kidney failure” OR “end-stage renal failure”)
Scopus (509)	TITLE-ABS-KEY ((Apixaban OR BMS562247 OR BMS-562247 OR Eliquis) AND ("antivitamins K" OR "vitamin K antagonists" OR warfarin OR coumadin OR phenprocoumon OR phenprocoumarol) AND (stroke OR "cerebrovascular accident" OR hemorrhage OR bleeding OR epistaxis OR hematemesis OR melena) AND "Atrial Fibrillation" AND ("chronic renal insufficiency" OR "chronic kidney insufficiency" OR "chronic kidney disease" OR "chronic renal disease" OR "end-stage kidney disease" OR "end-stage renal disease" OR "end-stage kidney failure" OR "end-stage renal failure"))
ScienceDirect (1,831)	Apixaban AND (warfarin OR phenprocoumon) AND (stroke OR hemorrhage OR bleeding) AND "Atrial Fibrillation" AND ("chronic kidney disease" OR "end-stage renal disease")
Cochrane Library (32)	(Apixaban OR BMS562247 OR BMS-562247 OR Eliquis) AND ("antivitamins K" OR "vitamin K antagonists" OR warfarin OR coumadin OR phenprocoumon OR phenprocoumarol) AND (stroke OR "cerebrovascular accident" OR hemorrhage OR bleeding OR epistaxis OR hematemesis OR melena) AND "Atrial Fibrillation" AND ("chronic renal insufficiency" OR "chronic kidney insufficiency" OR "chronic kidney disease" OR “chronic renal disease” OR "end-stage kidney disease" OR "end-stage renal disease" OR "end-stage kidney failure" OR "end-stage renal failure")
EBSCO Open Dissertations (20)	Apixaban AND warfarin AND (stroke OR bleeding) AND "Atrial Fibrillation" AND ("chronic kidney disease" OR "end-stage renal disease")

Table S2. Intervention and comparator regimens

Author, year	Apixaban regimen, N (%)	Dose reduction criteria	VKA comparator	INR target range and TTR (%)
Siontis 2018	- 2.5 mg BID: 1,317 (56.0)<br>- 5 mg BID: 1,034 (44.0)	N/A	Warfarin	N/A
Stanifer 2020	- 2.5 mg BID: 88 (64.7)<br>- 5 mg BID: 48 (35.3)	≥2 of the following criteria:<br>(1) age ≥80 years;<br>(2) body weight ≤60 kg;<br>(3) serum creatinine ≥1.5 mg/dL	Warfarin	- INR target range: 2-3<br>- The mean TTR: 55%
Elis 2021	2.5 mg BID: 76 (100)	Fixed dose 2.5 mg BID for all patients; no individual dose reduction criteria were specified.	Warfarin	N/A
Fu CM 2021	- Reduced-dose 2.5-5 mg/day: 915 (56.3)<br>- Standard dose 10 mg/day: 710 (43.7)	N/A	Warfarin	- The mean INR was 1.97±1.01 during the total follow-up period<br>- TTR: N/A
Wetmore 2022	- Label-concordant apixaban (5 mg BID): 2,382 (13.9)<br>- Below-label apixaban (2.5 mg BID, when 5 mg was indicated): 2,257 (13.2)	≥2 of the following criteria:<br>(1) age ≥80 years;<br>(2) body weight ≤60 kg;<br>(3) serum creatinine ≥1.5 mg/dL	Warfarin	N/A
Reinecke 2023	2.5 mg BID: 48 (100)	Fixed dose 2.5 mg BID for all patients; no individual dose reduction criteria were specified.	Phenprocoumon	- INR target range: 2-3<br>- The median TTR: 50.7% (range 0 to 100%)
Xu 2023	- 5 mg BID: 1,705 (40)<br>- 2.5 mg BID: 2,608 (60)	N/A	N/A[a]	N/A
Fu EL 2024	- 5 mg: N= 2,060<br>- 2.5 mg: N= 4,191<br>(after 1:1 propensity score matching)	N/A	Warfarin	N/A
Wu 2025	- Pure 2.5 mg: N= 237<br>- Pure 5 mg: N= 314<br>- Pure unknown dose: N= 285<br>- Mixed dose: N = 528	N/A	Warfarin	- INR target range: N/A<br>- The mean TTR: 44.4%

[a] This study compared apixaban dose groups without a VKA comparator.
INR: international normalized ratio, N/A: not applicable, TTR: time in the therapeutic range, VKA: vitamin K antagonist

Table S3. Comorbidities and concomitant medications of included studies

Comorbidities, N (%)	Siontis 2018	Stanifer 2020	Elis 2021	Fu CM 2021	Wetmore 2022 [a]	Reinecke 2023	Xu 2023 [a]	Fu EL 2024	Wu 2025
Anemia	25,336 (99.3)	N/A	N/A	N/A	N/A	N/A	N/A	7,997 (64.0)	N/A
Cancer	3,848 (15.1)	N/A	N/A	351 (10.8)	8.2%	N/A	N/A	2,655 (21.3)	N/A
Congestive heart failure	19,827 (77.7)	131 (48.7)	96 (63.2)	1,056 (32.5)	65.1%	N/A	64.9%	inpatient: 6,286 (50.3) outpatient: 7,934 (63.5)	N/A
Coronary artery disease	N/A	91 (34.0)	77 (50.7)	N/A	N/A	65 (67.0)	68.8%	8,275 (66.3)	1,075 (39.4)
Diabetes mellitus	19,121 (74.9)	59 (21.9)	60 (39.5)	947 (29.1)	76.8%	N/A	55.7%	8,115 (65.0)	1,400 (51.3)
Heart valve disease	N/A	N/A	N/A	N/A	22.0%	46 (47.4)	N/A	N/A	N/A
History of falls	N/A	N/A	15 (9.9)	N/A	11.7%	N/A	N/A	1,218 (9.8)	N/A
Hypertension	25,421 (99.6)	237 (88.1)	95 (62.5)	2,363 (72.71)	95.7%	N/A	95.2%	12,229 (97.9)	2,284 (83.7)
Liver dysfunction	2,580 (10.1)	N/A	3 (2.0)	244 (7.5)	12.9%	N/A	N/A	1,321 (10.6)	N/A
Myocardial infarction	6,850 (26.8)	N/A	N/A	189 (5.8)	26.2%	21 (21.7)	N/A	1,778 (14.2)	N/A
Peripheral vascular disease	11,521 (45.1)	42 (15.8)	N/A	73 (2.3)	35.9%	N/A	N/A	3,391 (27.2)	N/A
**Concomitant Medications, N (%)**	**Siontis 2018**	**Stanifer 2020**	**Elis 2021**	**Fu CM 2021**	**Wetmore 2022**	**Reinecke 2023**	**Xu 2023**	**Fu EL 2024**	**Wu 2025**
Antiarrhythmics	5,616 (22.0)	Amiodarone: 29 (11.0) Digoxin: 95 (36.0)	N/A	Amiodarone: 603 (18.6) Digoxin: 295 (9.1)	21.2%	N/A	N/A	2,634 (21.1)	1,480 (54.3)
Anti-hypertension	ACEIs: 3,195 (12.5) ARBs: 1,474 (5.8) CCBs: 5,946 (23.3)	ACEIs or ARBs: 173 (65.5)	N/A	2,508 (77.2)	ACEIs or ARBs: 28.7%	ACEIs or ARBs: 37 (38.1)	ACEIs: 12.4% ARBs: 13.1% CCBs: 18.3%	ACEIs or ARBs: 4,484 (35.9) CCBs: 692 (5.5)	ACEIs: 551 (20.2) ARBs:

	BBs: 10,645 (41.7)<br>Diuretic: 2,329 (9.1)	CCBs: 90 (34.1)<br>BBs: 136 (51.5)			BBs: 61.3%	CCBs: 21 (21.7)<br>BBs: 76 (78.4)<br>Diuretic: 64 (66.0)	BBs: 19.6%<br>Diuretic: 33.2%	BBs: 9,951 (79.7)<br>Diuretic: 10,177 (81.5)	490 (18.0)<br>BBs: 1,867 (68.4)
Anti-platelet agent	1,866 (7.3);<br>Clopidogrel (94.4%),<br>Prasugrel (2%),<br>Ticagrelor (1.7%),<br>Dipyridamole (1.7%),<br>Ticlopidine (0.2%)	Aspirin: 99 (36.8)<br>Clopidogrel: 5 (1.9)	N/A	1,398 (43.0);<br>Aspirin: 1,095 (33.7),<br>Clopidogrel: 409 (12.6),<br>Ticagrelor: 14 (0.4)	17.0%	Aspirin: 33 (34.0)	P_2Y_{12} inhibitor: 8.7%<br>Aspirin: 5.7%<br>Dual antiplatelet therapy: 2.9%	3,063 (24.5)	N/A
Gastric antacids	PPI: 5,036 (19.7)	86 (32.6)	N/A	844 (26.0)	N/A	N/A	N/A	PPI: 5,086 (40.7)<br>H2 blockers: 1443 (11.6)	N/A
Glucose-lowering agent	Insulin: 3,419 (13.4)	-	N/A	682 (21.0)	29.1%	SGLT2i: 0 (0)	Insulin: 17.5%<br>Sulfonylureas: 11.1%<br>Metformin: 5.7%<br>SGLT2i: 0.6%<br>GLP-1 RA: 1.9%	Insulin: 3,319 (26.6)<br>Metformin: 856 (6.9)<br>Sulfonylurea: 2,375 (19.0)	N/A
Lipid-lowering agent	Statin: 6,174 (24.2)	Statin: 108 (40.9)	N/A	817 (25.1)	Statin: 46.1%	Statin: 50 (51.6)	Statin: 25.4%	Statin: 9,470 (75.8)	N/A
NSAIDs	357 (1.4)	33 (12.5)	N/A	374 (11.5)	1.5%	N/A	5.1%	805 (6.4)	N/A

[a] Estimated by the authors based on available data.

ACEIs: angiotensin-converting enzyme inhibitors, ARBs: angiotensin II receptor blockers, BBs: beta-blockers, CCBs: calcium channel blockers, GLP-1 RA: glucagon-like peptide-1 receptor agonist, H2 blockers: histamine-2 receptor antagonists, N/A: not applicable, NSAIDs: non-steroidal anti-inflammatory drugs, PPI: proton pump inhibitor, SGLT2i: sodium-glucose cotransporter-2 inhibitor

Table S4. Definitions of outcomes

Author, year	Stroke/SE	Major Bleeding	Any bleeding	Mortality
Siontis 2018	Defined using ICD-9/10 codes as reported by Siontis et al.	Major bleeding is defined as a bleeding event with (i) a critical site code (intracranial, retroperitoneal, intraspinal, intra-ocular, pericardial, or intraarticular), (ii) a transfusion of blood products, or (iii) death, as described in Cunningham et al.	N/A	Mortality data were obtained from the United States Renal Data System (USRDS).
Stanifer 2020	N/A	Defined according to the International Society on Thrombosis and Haemostasis (ISTH) criteria.	Defined by the authors as a composite of major and clinically relevant non-major bleeding.	All-cause mortality
Elis 2021	N/A	Defined as fatal bleeding and/or symptomatic bleeding in a critical area or organ and/or bleeding leading to transfusion of two or more units of red cells	N/A	Mortality
Fu CM 2021	Defined using ICD-9/10 codes as reported by Fu CM et al.	Defined using ICD-9/10 codes as reported by Fu CM et al.	N/A	In-hospital all-cause mortality
Wetmore 2022	Defined using ICD-9/10 codes as reported by Wetmore et al.	Major bleeding was defined to include bleeding events that were (1) fatal, and/or (2) involved a critical site, and/or (3) required a blood transfusion. (Cunningham bleeding algorithm)	N/A	All-cause mortality
Reinecke 2023	N/A	Defined according to the International Society on Thrombosis and Haemostasis (ISTH) criteria.	Defined by the authors as a composite of major and clinically relevant non-major bleeding.	All-cause mortality
Xu 2023	Defined using ICD-9/10 codes as reported by Xu et al.	Defined using ICD-9/10 codes as reported by Xu et al.	Defined using ICD-9/10 codes as reported by Xu et al.	All-cause mortality
Fu EL 2024	Defined using ICD-9/10 codes as reported by Fu EL et al.	Defined using ICD-9/10 codes as reported by Fu EL et al.	N/A	All-cause mortality
Wu 2025	Defined using ICD-10 and CPT codes as reported by Wu et al.	Defined by the authors as bleeding events requiring blood transfusion.	Any bleeding was defined according to Wu et al. as all bleeding-related side effects, including gastrointestinal bleeding and bleeding events requiring blood transfusion.	All-cause mortality

CPT: Current Procedural Terminology, ICD: International Classification of Diseases, ISTH: International Society on Thrombosis and Haemostasis, N/A: not applicable, SE: systemic embolism

Table S5. Network meta-analysis estimates for stroke or systemic embolism, major bleeding, any bleeding, and all-cause mortality

**Outcomes**	**Comparisons**		
	**Standard-dose apixaban vs Reduced-dose apixaban**	**Standard-dose apixaban vs VKAs**	**Reduced-dose apixaban vs VKAs**
**Stroke/SE**			
HR (95% CI)	1.05 (0.66-1.68)	0.59 (0.39-0.89) *	0.56 (0.37-0.85) *
**Major bleeding**			
HR (95% CI)	0.95 (0.81-1.12)	0.54 (0.47-0.61) *	0.56 (0.50-0.64) *
**Any bleeding**			
HR (95% CI)	1.07 (0.90-1.28)	0.63 (0.54-0.73) *	0.59 (0.51-0.67) *
**All-cause mortality**			
HR (95% CI)	0.73 (0.62-0.87) *	0.55 (0.47-0.64) *	0.75 (0.65-0.86) *

* Statistically significant difference (p-value < 0.05),
CI: confidence interval, HR: hazard ratio, SE: systemic embolism, VKAs: vitamin K antagonists

Table S6. Surface under the cumulative ranking curve (SUCRA) values

- Table S6.1 Stroke or systemic embolism

Treatment	SUCRA	PrBest	MeanRank
1.Vitamin K antagonists (VKAs)	0.6	0.0	3.0
2.Reduced-dose apixaban	78.3	57.2	1.4
3.Standard-dose apixaban	71.1	42.8	1.6

- Table S6.2 Major bleeding

Treatment	SUCRA	PrBest	MeanRank
1.Vitamin K antagonists (VKAs)	0.0	0.0	3.0
2.Reduced-dose apixaban	62.7	25.4	1.7
3.Standard-dose apixaban	87.3	74.6	1.3

- Table S6.3 Any bleeding

Treatment	SUCRA	PrBest	MeanRank
1.Vitamin K antagonists (VKAs)	0.0	0.0	3.0
2.Reduced-dose apixaban	89.2	78.4	1.2
3.Standard-dose apixaban	60.8	21.6	1.8

- Table S6.4 All-cause mortality

Treatment	SUCRA	PrBest	MeanRank
1.Vitamin K antagonists (VKAs)	0.0	0.0	3.0
2.Reduced-dose apixaban	50.0	0.0	2.0
3.Standard-dose apixaban	100.0	100.0	1.0

Table S7. Confidence in Network Meta-Analysis (CINeMA) assessment

- Table S7.1 Stroke or systemic embolism

Comparison	Number of studies	Within-study bias	Reporting bias	Indirectness	Imprecision [a]	Heterogeneity	Incoherence	Confidence rating	Reason(s) for downgrading
Reduced dose apixaban vs Standard dose apixaban	2	Some concerns	Low risk	No concerns	Major concerns	No concerns	No concerns	Very low	Within-study bias, Imprecision
Reduced dose apixaban vs VKAs	5	Some concerns	Low risk	No concerns	No concerns	Some concerns	No concerns	Low	Within-study bias, Heterogeneity
Standard dose apixaban vs VKAs	4	Some concerns	Low risk	No concerns	No concerns	Major concerns	No concerns	Very low	Within-study bias, Heterogeneity

- Table S7.2 Major bleeding

Comparison	Number of studies	Within-study bias	Reporting bias	Indirectness	Imprecision [a]	Heterogeneity	Incoherence	Confidence rating	Reason(s) for downgrading
Reduced dose apixaban vs Standard dose apixaban	2	Some concerns	Low risk	No concerns	Major concerns	No concerns	No concerns	Very low	Within-study bias, Imprecision
Reduced dose apixaban vs VKAs	5	Some concerns	Low risk	No concerns	No concerns	Major concerns	No concerns	Very low	Within-study bias, Heterogeneity
Standard dose apixaban vs VKAs	4	Some concerns	Low risk	No concerns	No concerns	Major concerns	No concerns	Very low	Within-study bias, Heterogeneity

- Table S7.3 Any bleeding

Comparison	Number of studies	Within-study bias	Reporting bias	Indirectness	Imprecision [a]	Heterogeneity	Incoherence	Confidence rating	Reason(s) for downgrading
Reduced dose apixaban vs Standard dose apixaban	2	Some concerns	Low risk	No concerns	Major concerns	No concerns	No concerns	Very low	Within-study bias, Imprecision
Reduced dose apixaban vs VKAs	4	Some concerns	Low risk	No concerns	No concerns	No concerns	No concerns	Moderate	Within-study bias
Standard dose apixaban vs VKAs	3	Some concerns	Low risk	No concerns	No concerns	No concerns	No concerns	Moderate	Within-study bias

- Table S7.4 All-cause mortality

Comparison	Number of studies	Within-study bias	Reporting bias	Indirectness	Imprecision [a]	Heterogeneity	Incoherence	Confidence rating	Reason(s) for downgrading
Reduced dose apixaban vs Standard dose apixaban	2	Some concerns	Low risk	No concerns	No concerns	Some concerns	No concerns	Low	Within-study bias, Heterogeneity
Reduced dose apixaban vs VKAs	5	Some concerns	Low risk	No concerns	No concerns	Some concerns	No concerns	Low	Within-study bias, Heterogeneity
Standard dose apixaban vs VKAs	3	Some concerns	Low risk	No concerns	No concerns	No concerns	No concerns	Moderate	Within-study bias

[a] Thresholds were derived from 1-year age- and sex-adjusted standardized risks reported by Ballegaard et al [1]. Absolute risk differences between OAC and no OAC were used to estimate the minimal clinically important difference (MCID) and corresponding equivalence margins on the ratio scale.

1. Ballegaard ELF, Olesen JB, Kamper AL, Feldt-Rasmussen B, Gislason G, Torp-Pedersen C, et al. Oral anticoagulation for stroke prevention in atrial fibrillation and advanced kidney disease. Res Pract Thromb Haemost. 2024;8(2):102350.

Table S8. Sensitivity analysis excluding studies reporting crude event data (pairwise meta-analysis)

Outcomes	Comparisons		
	**Standard-dose apixaban vs Reduced-dose apixaban**	**Standard-dose apixaban vs VKAs**	**Reduced-dose apixaban vs VKAs**
**Stroke/SE**			
HR (95% CI), I^2 value	0.78 (0.55-1.10), 45%	0.78 (0.64-0.93), 0%*	0.78 (0.58-1.05), 48%
**Major bleeding**			
HR (95% CI), I^2 value	1.08 (0.90-1.29), 0%	0.61 (0.53-0.70), 18%*	0.71 (0.63-0.81), 17%*
**Any bleeding**			
HR (95% CI), I^2 value	1.10 (0.47-2.58), 76%	0.65 (0.47-0.89), 0%*	0.51 (0.15-1.70), 90%
**All-cause mortality**			
HR (95% CI), I^2 value	0.91 (0.74-1.12), 59%	0.87 (0.76-1.00), 65%*	0.98 (0.89-1.07), 23%

* Statistically significant difference (p-value < 0.05),
CI: confidence interval, HR: hazard ratio, SE: systemic embolism, VKAs: vitamin K antagonists

Table S9. Subgroup analysis results from the pairwise meta-analysis

**Subgroup outcomes,** HR (95% CI), I^2 value	**Comparisons**		
	**Standard-dose apixaban vs Reduced-dose apixaban**	**Standard-dose apixaban vs VKAs**	**Reduced-dose apixaban vs VKAs**
**Randomised study**			
Stroke/SE	N/A	N/A	0.34 (0.01-9.71), N/A
Major bleeding	N/A	0.34 (0.09-1.29), N/A	0.54 (0.24-1.20), 21%
Any bleeding	N/A	0.51 (0.17-1.53), N/A	0.53 (0.14-1.94), 89%
All-cause mortality	N/A	N/A	0.77 (0.36-1.65), N/A
**Non-randomised study**			
Stroke/SE	0.78 (0.55-1.10), 45%	0.78 (0.65-0.94), 11%*	0.80 (0.60-1.06), 40%
Major bleeding	1.08 (0.90-1.29), 0%	0.62 (0.53-0.73), 35%*	0.72 (0.64-0.82), 16%*
Any bleeding	1.10 (0.47-2.58), 76%	0.66 (0.47-0.92), N/A*	0.92 (0.63-1.35), N/A
All-cause mortality	0.91 (0.74-1.12), 59%	0.87 (0.76-1.00), 65%*	0.96 (0.81-1.12), 68%
**On-dialysis**			
Stroke/SE	0.81 (0.48-1.36), 62%	0.79 (0.62-1.02), 35%	0.97 (0.78-1.21), 0%
Major bleeding	0.98 (0.79-1.22), 0%	0.68 (0.58-0.80), 0%*	0.70 (0.59-0.81), 0%*
Any bleeding	N/A	N/A	1.02 (0.56-1.85), N/A
All-cause mortality	0.78 (0.59-1.04), 61%	0.76 (0.57-1.01), 71%	0.98 (0.91-1.06), 0%
**Non-dialysis**			
Stroke/SE	1.01 (0.59-1.73), N/A	0.91 (0.61-1.36), 33%	0.63 (0.40-0.99), 0%*
Major bleeding	1.51 (0.83-2.74), N/A	0.53 (0.27-1.03), 25%	0.67 (0.54-0.84), 11%*
Any bleeding	1.63 (1.04-2.55), N/A*	0.51 (0.17-1.53), N/A	0.27 (0.14-0.52), N/A*
All-cause mortality	1.03 (0.77-1.38), N/A	0.94 (0.85-1.04), N/A	0.65 (0.31-1.34), 83%

* Statistically significant difference (p-value < 0.05)
CI: confidence interval, HR: hazard ratio, N/A: not applicable, SE: systemic embolism, VKAs: vitamin K antagonists

Table S10. Results of inconsistency testing in the network meta-analysis

Outcomes	Results of inconsistency testing
Stroke or systemic embolism	(1) [_y_B]des_BC = 0<br>chi2(1) = 0.21<br>Prob > chi2 = 0.6474
Major bleeding	(1) [_y_B]des_BC = 0<br>chi2(1) = 0.02<br>Prob > chi2 = 0.8759
Any bleeding	(1) [_y_B]des_BC = 0<br>chi2(1) = 1.50<br>Prob > chi2 = 0.2211
All-cause mortality	(1) [_y_B]des_BC = 0<br>chi2(1) = 1.39<br>Prob > chi2 = 0.2377

Table S11. Baseline characteristics of included studies relevant to the assessment of transitivity

Author, year	Study design	Male, N (%)	Age, years [a]	BW (kg) or BMI [a]	CKD severity	Dialysis status	CHA_2DS_2-VASc score [a]	HAS-BLED Score [a]	Prior stroke/SE, N (%)	Prior bleeding, N (%)	Antiplatelet use N (%)	Location
Siontis 2018	Retrospective cohort study	13,852 (54.3)	68.22 (11.89)	N/A	Stage 5	Dialysis	5.24 (1.79)	N/A	8,461 (33.2)	Major bleeding: 2,536 (9.9) GI bleeding: 2,966 (11.6)	1,866 (7.3); Clopidogrel (94.4%), Prasugrel (2%), Ticagrelor (1.7%), Dipyridamole (1.7%), Ticlopidine (0.2%)	United States
Stanifer 2020	Double-blind randomized controlled trial [b]	106 (39.4)	80.65 (6.71)	BW 57.00 (11.93)	Stage 4	Non-dialysis	4.80 (1.42)	2.30 (1.05)	73 (27.1)	N/A	Aspirin: 99 (36.8) Clopidogrel: 5 (1.9)	Multicenter study across 39 countries (North America, Latin America, Europe, Asia-Pacific)
Elis 2021	Sub-analysis of a multicenter prospective cohort study	81 (53.3)	80.58 (9.81)	N/A	Stage 4	Non-dialysis	5.13 (1.40)	2.49 (1.06)	18 (11.8)	12 (7.9)	N/A	Israel
Fu CM 2021	Retrospective cohort study	1,864 (57.4)	74-75 (NR)	N/A	Stage 4-5	Non-dialysis	3.82 (1.69)	2.92 (1.35)	1,030 (31.7)	963 (29.6)	1,398 (43.0); Aspirin: 1,095 (33.7), Clopidogrel: 409 (12.6), Ticagrelor: 14 (0.4)	Taiwan
Wetmore 2022	Retrospective cohort study	10,585 (61.7) [c]	66.20 (9.40)	N/A	Stage 5	Dialysis	4.50 (1.70)	3.00 (0.80)	21.2% [c]	N/A	17.0%	United States
Reinecke 2023	Prospective randomized open blinded endpoint	68 (70.1)	74.70 (7.90)	BMI 28.60 (6.10)	Stage 5	Dialysis	4.52 (1.55)	4.20 (1.02)	N/A	N/A	Aspirin: 33 (34.0)	Germany
Xu 2023	Retrospective cohort study	43.0% [c]	77.00 (9.00)	BW 87.00	Stage 4-5	Non-dialysis	3.70 (1.60)	2.50 (1.00)	34.4% [c]	3.8% [c]	P_2Y_{12} inhibitor: 8.7% Aspirin: 5.7%	United States

				(24.50)							Dual antiplatelet therapy: 2.9%	
Fu EL 2024	Retrospective cohort study	6,324 (50.6)	78.75 (7.60)	N/A	Stage 4-5	Non-dialysis	5.38 (1.50)	2.91 (0.67)	3,546 (28.40)	3,400 (27.23)	3,063 (24.5)	United States
Wu 2025	Retrospective cohort study	1,626 (59.6)	70.40 (10.50)	BMI 30.65 (7.10)	Stage 5	Dialysis and non-dialysis	N/A	N/A	464 (17.0)	N/A	N/A	TriNetX Global Collaborative Network, 104 health care organizations in 15 countries

[a] Data reported as mean (SD) unless otherwise specified., [b] Subgroup analysis of the ARISTOTLE trial in patients with CrCl 25-30 mL/min., [c] Estimated by the authors based on available data.

BMI: body mass index, BW: body weight, CKD: chronic kidney disease, CrCl: creatinine clearance , N/A: not applicable, SD: standard deviation, SE: systemic embolism

Figure S1. Risk of bias assessment of randomized controlled trials using the Cochrane Risk of Bias 2 (RoB 2) tool

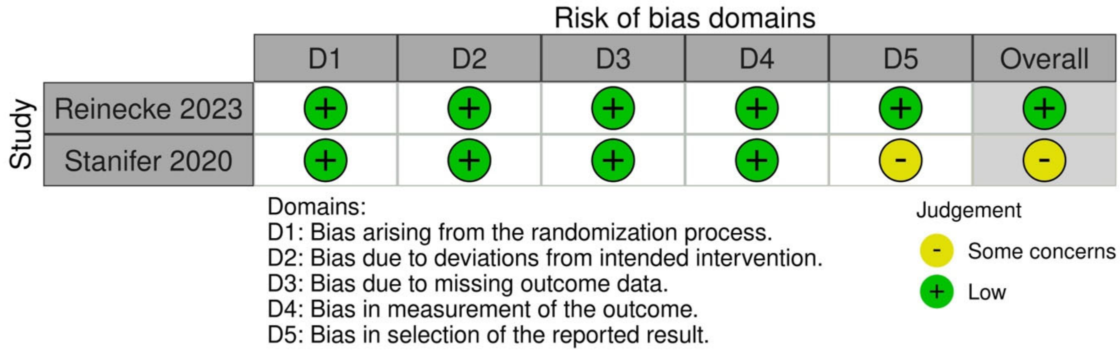

Figure S2. Risk of bias assessment of non-randomized studies using the ROBINS-I tool

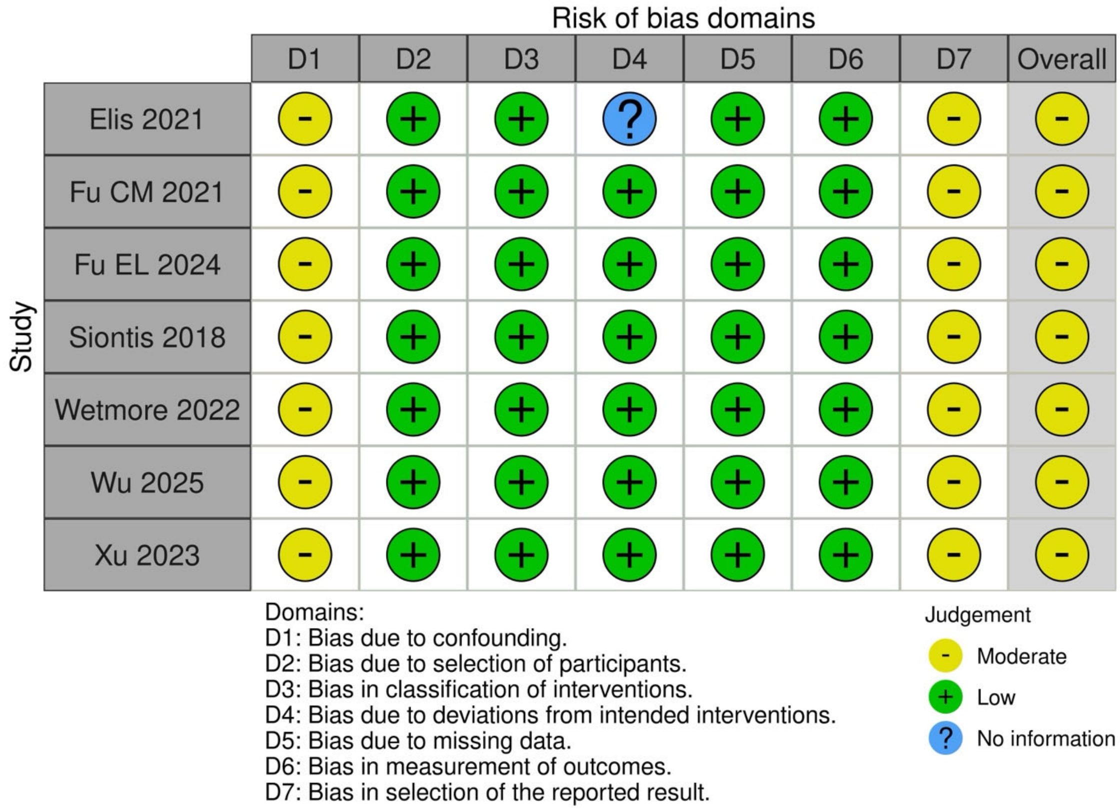

Figure S3. Surface under the cumulative ranking curve (SUCRA) plots for treatment ranking

- Figure S3.1 Stroke or systemic embolism

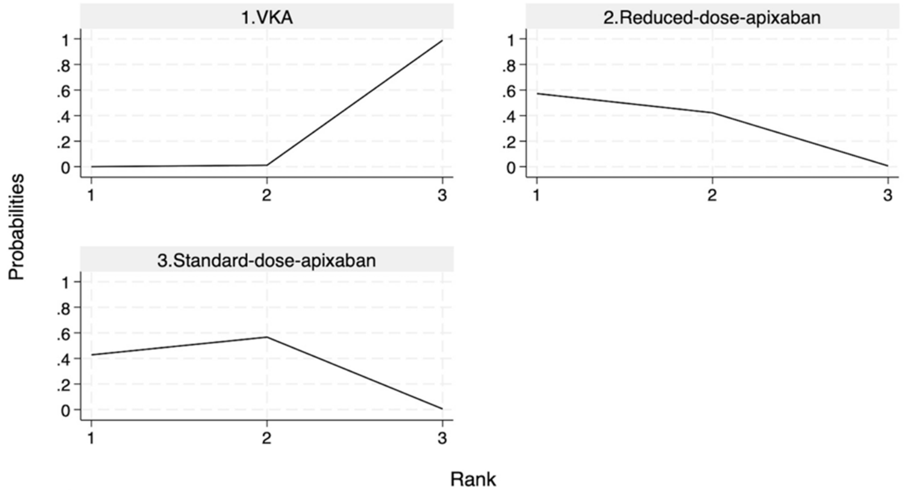

- Figure S3.2 Major bleeding

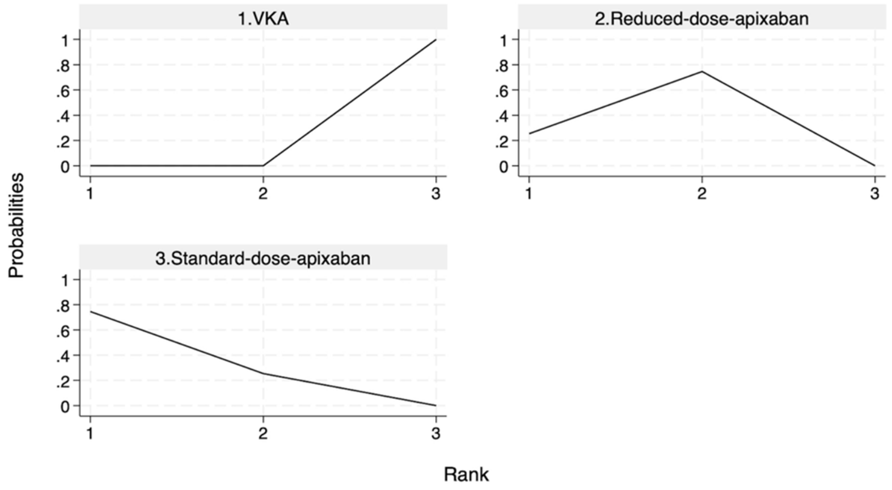

- Figure S3.3 Any bleeding

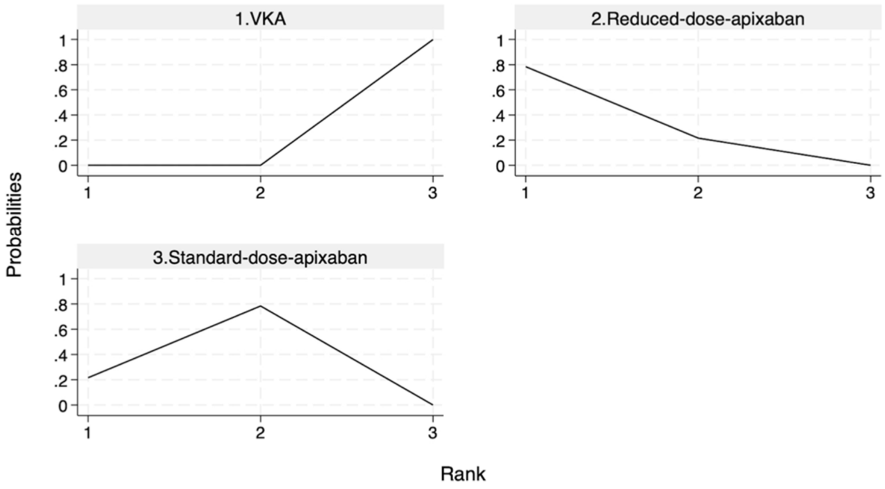

- Figure S3.4 All-cause mortality

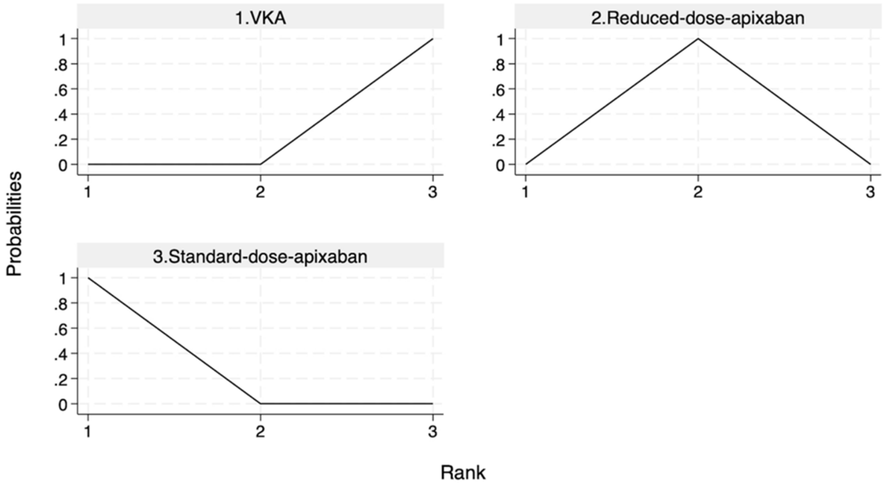

Figure S4. Forest plots from pairwise meta-analyses comparing treatment regimens

- Figure S4.1 Stroke or systemic embolism comparing

  (A) standard-dose apixaban versus reduced-dose apixaban,

  (B) standard-dose apixaban versus VKAs, and

  (C) reduced-dose apixaban versus VKAs

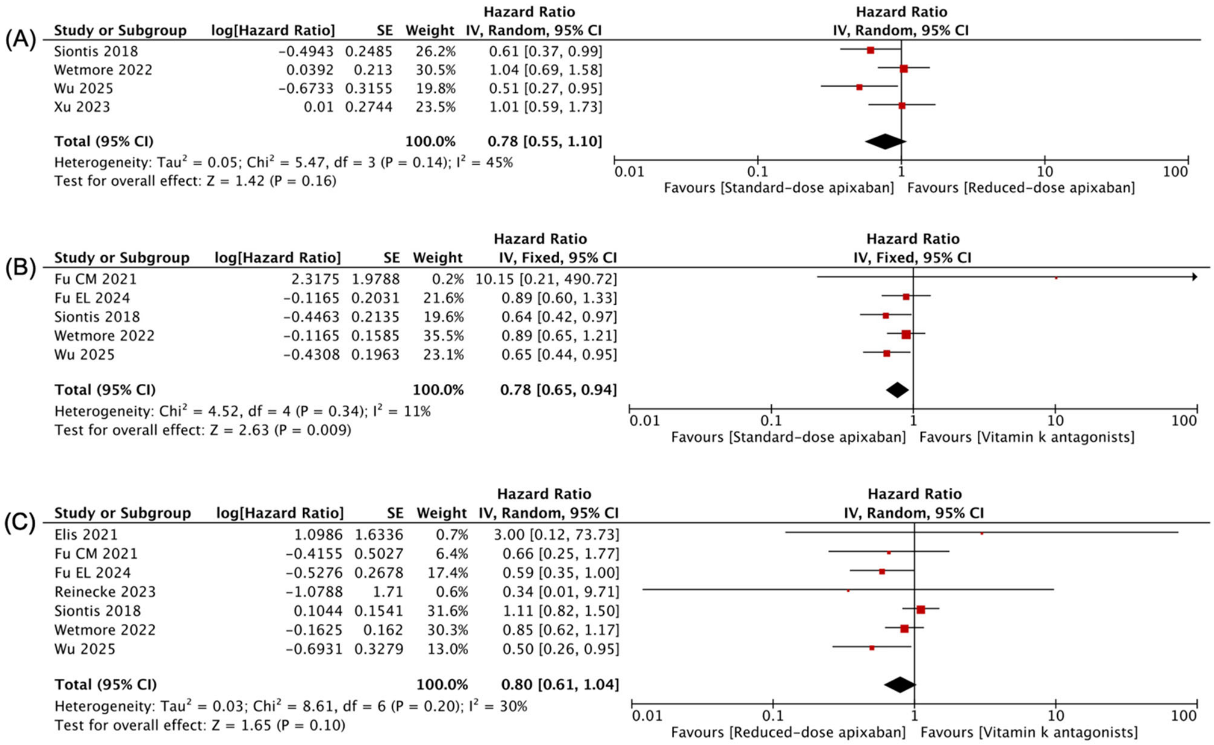

- Figure S4.2 Major bleeding comparing

  (A) standard-dose apixaban versus reduced-dose apixaban,

  (B) standard-dose apixaban versus VKAs, and

  (C) reduced-dose apixaban versus VKAs

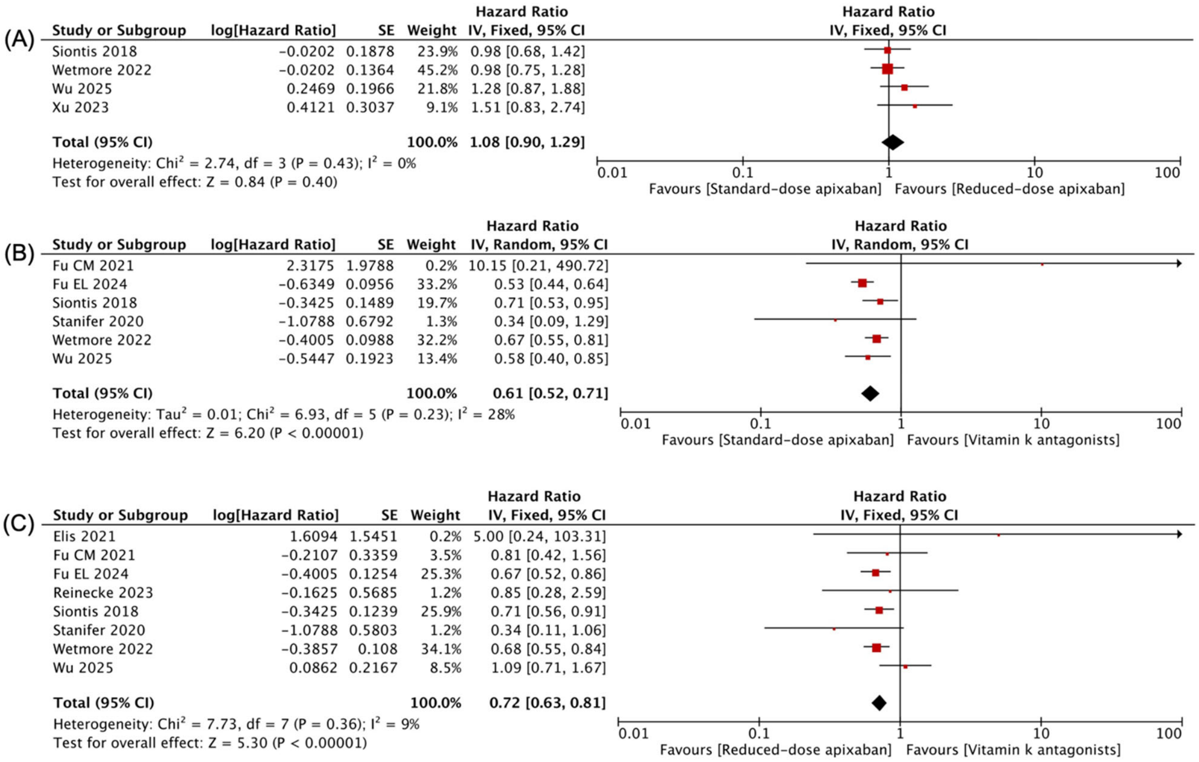

- Figure S4.3 Any bleeding comparing
  (A) standard-dose apixaban versus reduced-dose apixaban,
  (B) standard-dose apixaban versus VKAs, and
  (C) reduced-dose apixaban versus VKAs

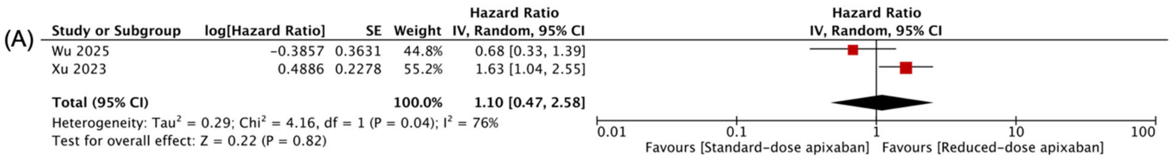

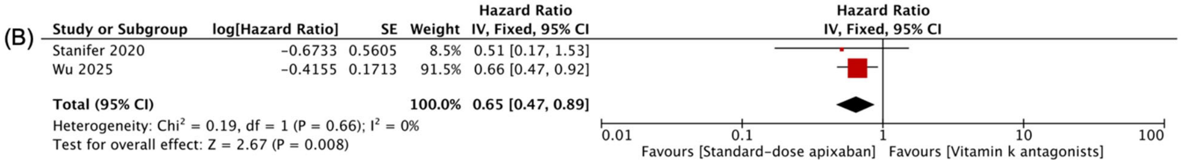

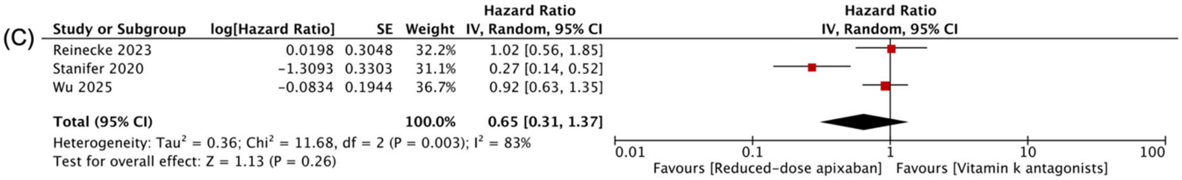

Figure S4.4 All-cause mortality comparing

(A) standard-dose apixaban versus reduced-dose apixaban,

(B) standard-dose apixaban versus VKAs, and

(C) reduced-dose apixaban versus VKAs

(A)

Study or Subgroup	log[Hazard Ratio]	SE	Weight	Hazard Ratio IV, Random, 95% CI
Siontis 2018	−0.4463	0.1824	19.1%	0.64 [0.45, 0.92]
Wetmore 2022	−0.1393	0.0582	38.8%	0.87 [0.78, 0.98]
Wu 2025	0.207	0.187	18.6%	1.23 [0.85, 1.77]
Xu 2023	0.0296	0.1488	23.5%	1.03 [0.77, 1.38]
**Total (95% CI)**			**100.0%**	**0.91 [0.74, 1.12]**

Heterogeneity: $Tau^2 = 0.03$; $Chi^2 = 7.38$, df = 3 (P = 0.06); $I^2 = 59\%$
Test for overall effect: Z = 0.89 (P = 0.38)

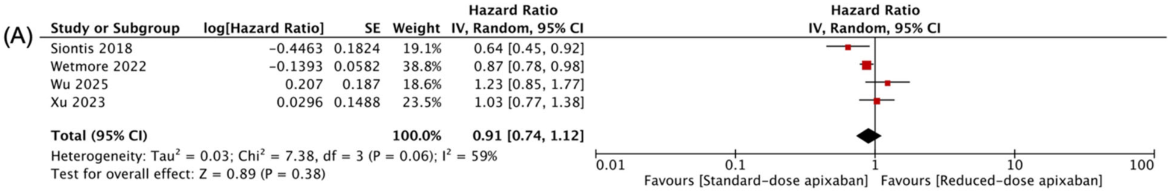

(B)

Study or Subgroup	log[Hazard Ratio]	SE	Weight	Hazard Ratio IV, Random, 95% CI
Fu EL 2024	−0.0619	0.0515	36.7%	0.94 [0.85, 1.04]
Siontis 2018	−0.462	0.1566	13.6%	0.63 [0.46, 0.86]
Wetmore 2022	−0.1625	0.0421	39.4%	0.85 [0.78, 0.92]
Wu 2025	0.1222	0.188	10.4%	1.13 [0.78, 1.63]
**Total (95% CI)**			**100.0%**	**0.87 [0.76, 1.00]**

Heterogeneity: $Tau^2 = 0.01$; $Chi^2 = 8.64$, df = 3 (P = 0.03); $I^2 = 65\%$
Test for overall effect: Z = 1.99 (P = 0.05)

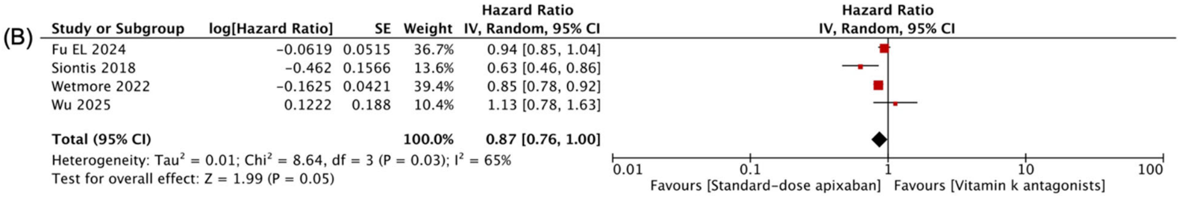

(C)

Study or Subgroup	log[Hazard Ratio]	SE	Weight	Hazard Ratio IV, Random, 95% CI
Elis 2021	−0.8675	0.3007	5.6%	0.42 [0.23, 0.76]
Fu EL 2024	−0.1165	0.0767	26.1%	0.89 [0.77, 1.03]
Reinecke 2023	−0.2614	0.3884	3.6%	0.77 [0.36, 1.65]
Siontis 2018	0.0677	0.1083	20.8%	1.07 [0.87, 1.32]
Wetmore 2022	−0.0305	0.0422	31.8%	0.97 [0.89, 1.05]
Wu 2025	0.27	0.1809	12.1%	1.31 [0.92, 1.87]
**Total (95% CI)**			**100.0%**	**0.95 [0.82, 1.11]**

Heterogeneity: $Tau^2 = 0.02$; $Chi^2 = 12.87$, df = 5 (P = 0.02); $I^2 = 61\%$
Test for overall effect: Z = 0.66 (P = 0.51)

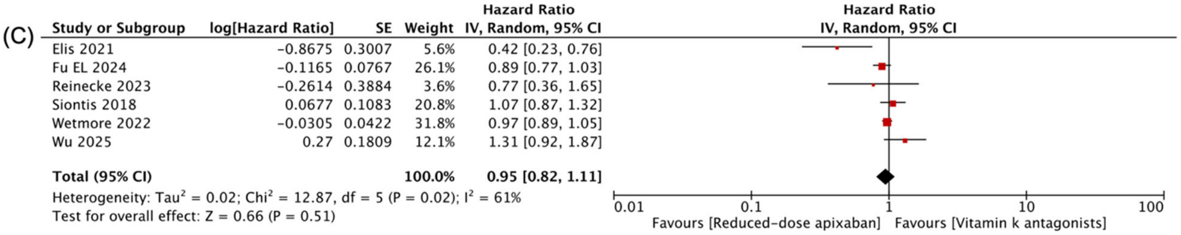

Figure S5. Comparison-adjusted funnel plots from the network meta-analysis

- Figure S5.1 Stroke or systemic embolism

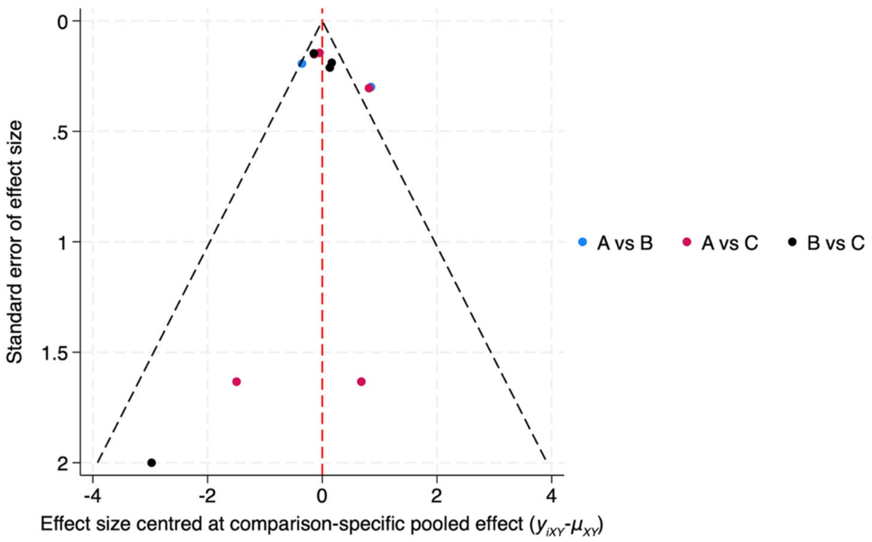

- Figure S5.2 Major bleeding

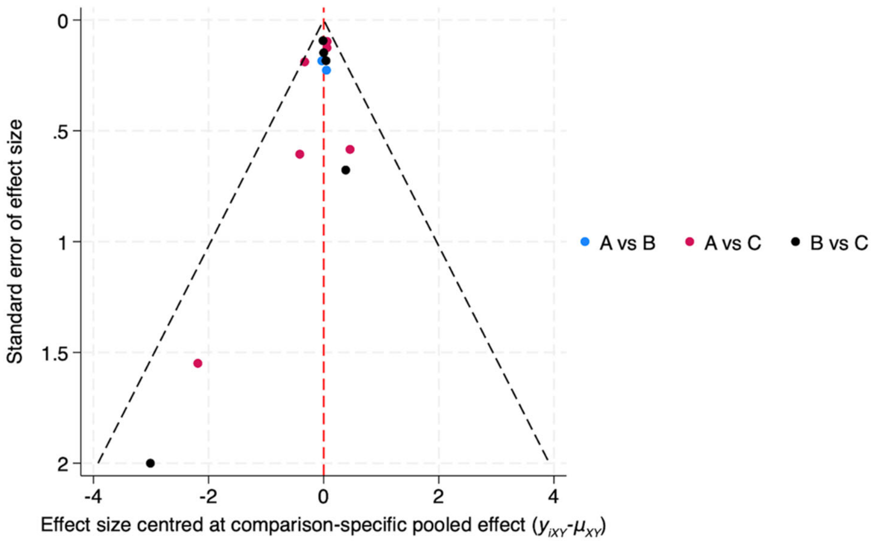

- Figure S5.3 Any bleeding

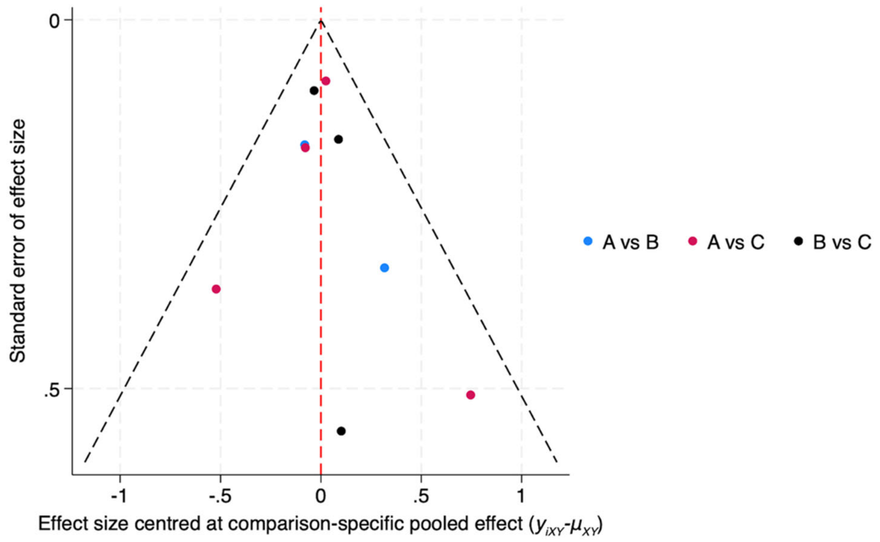

- Figure S5.4 All-cause mortality

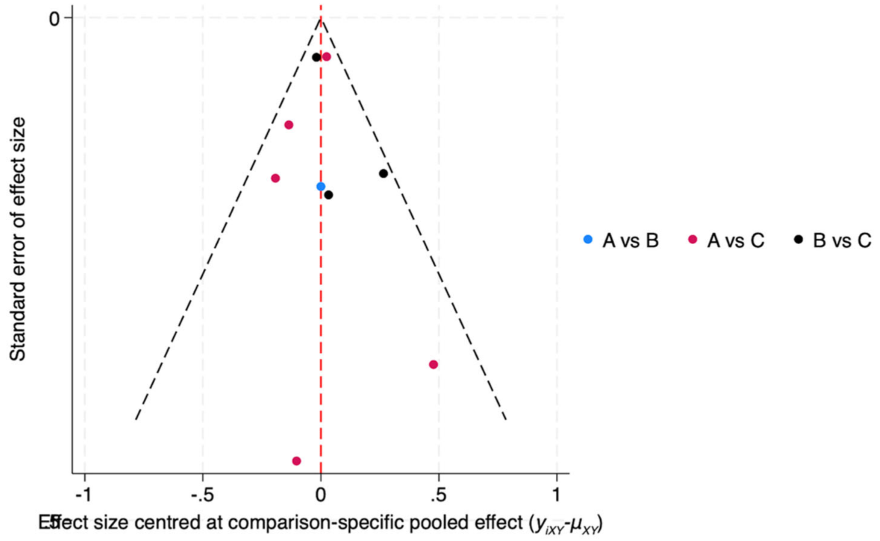

Figure S6. Funnel plot of pairwise meta-analyses

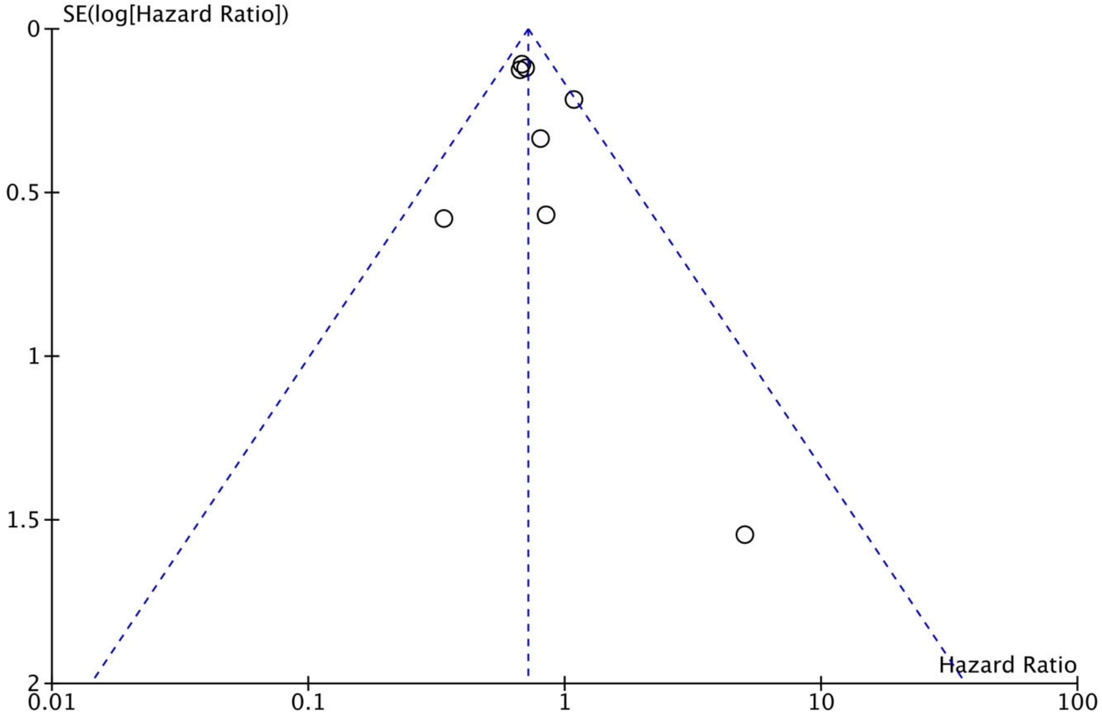

Supplement: Supplementary file 1 [file jcm-15-04664-s001.zip › jcm-4334558 - File S2.pdf]
